# Assessing the predictive power of psychological empowerment and health literacy for older patients’ participation in health care: a cross-sectional population-based study

**DOI:** 10.1186/s12877-017-0448-x

**Published:** 2017-02-20

**Authors:** Gabriele Sak, Fabia Rothenfluh, Peter J. Schulz

**Affiliations:** Institute of Communication and Health (ICH), Università della Svizzera italiana (USI), Via G. Buffi 13, 6900 Lugano, Switzerland

**Keywords:** Older adults’ involvement, Shared decision-making, Health literacy, Empowerment, Treatment decision-making, Survey, Switzerland

## Abstract

**Background:**

Research has confirmed a positive link between patient involvement in decision-making and improvements in health outcomes. The objective of this study was to examine the roles of psychological empowerment and health literacy on the elderly’s willingness to engage in treatment decisions.

**Methods:**

A self-administered questionnaire was completed by a randomly selected sample of Swiss adults aged 65–80 years old (*N* = 826). Multivariate logistic regression was applied to determine the contribution of health literacy, psychological empowerment, and trust in physician on participants’ preference to be active, collaborative or passive in decision-making.

**Results:**

Most of the survey respondents preferred some participation in dealing with health related decisions (collaborative: 51%, and active: 35.6%). More than two-thirds of the sample was satisfied with their current involvement in medical decision-making (72.7%). Roughly one-fifth (18.8%) wished to attain a more active engagement than currently experienced, and the remainder of the sample preferred the opposite (8.5%). Due to higher reported levels of psychological empowerment and health literacy, Swiss-German seniors significantly preferred and assumed higher participation in medical decisions than Swiss-Italians. Psychological empowerment correlated with older adults’ preferred and perceived involvement in medical decision-making. However, health literacy only predicted actual involvement in the last treatment decision that had to be made, differentiating only the active from the passive involvement group. Additionally, this research showed that health literacy mediated the relationship between psychological empowerment and the actual involvement in the last treatment decision that had to be made by the participant. Trust in physician and age appeared to be barriers to involvement, whereas education served as a facilitator.

**Conclusions:**

As older adults’ health literacy plays a role in individuals’ willingness to attain an active role in health care decision-making, public health efforts should aim at developing programs and appropriate information that facilitate this process, especially for individuals with moderate or lower levels of health literacy. The current investigation showed that adequate health literacy levels are essential (but not sufficient) in order to reach higher rates of participation in the healthcare context. This research complements past evidence by adding knowledge on the psychosocial antecedents, and their combined effects on patients’ involvement in healthcare.

## Background

The Swiss Federal Statistical Office predicts that in 2017, the Swiss population will consist of more individuals older than 64, than children and adolescents [1]. A good relationship with the physician that meets older people’s needs is therefore a topic of concern. With regard to doctor-patient relationships, older adults have experienced a major shift in the past decades. Traditionally, doctors were expected to decide for patients, and patients were expected to be passive recipients of medical care [2]. Recently, however, patients have been increasingly encouraged to play an active and autonomous role in healthcare and specifically in medical decision-making [3–5]. This socio-political change towards patient autonomy fosters patient empowerment. At the same time, it creates new demands on patients and may be particularly challenging for older patients, who have learned how to interact with their healthcare provider when paternalistic decision-making was the rule.

The positive aspects of involving patients in the management of their health have been documented (e.g., [6–8]). For example, involving patients in the decision-making process allows health providers to obtain specific information about beliefs, values, and preferences that would not be available with a one-way communication style [9]. Chewning and colleagues conducted a systematic review including 115 studies that tested patients’ preferences of engagement in the medical context. They found that the proportion of patients willing to participate more actively has increased over time [10]. However, evidence also shows that preferences for participation in healthcare decisions are highly inconsistent in general [11–14] and for older patients in particular [9, 15].

Previous research has identified personal and contextual characteristics (situation-specific) that are associated with patients’ decision-making preferences. To date, there is no clear picture of which psychological predictors influence the willingness to participate. The majority of studies focused on socio-demographic predictors for participation preferences (e.g., [11, 16, 17]): Age seems to matter in terms of willingness to participate in treatment decision-making. The majority of young patients want to engage in shared decision-making but this preference declines with increasing age of patients [5, 18, 19]. Nevertheless, a review on age-related changes in patients with cancer’s preferences for participation in treatment decisions showed mixed results [20]. The same authors attribute these findings partially to differences in the methods that were used to assess preferences, and partly to differences between samples (i.e., stage of the disease and socio-demographic characteristics). In addition, as the generation of elderly that participated in those studies had experienced major changes in health care over the decades (see f. ex. [21]), such results should be considered with caution due to potential cohort effects based on age-related differences.

With respect to contextual characteristics, the type of participation seems to matter: patients want to be involved in decisions that do not require medical expertise, but not as much in decisions that require medical knowledge [22]. Other studies showed that the severity of the illness predicts patients’ willingness to engage in shared decision-making [23–26]. The more the disease is considered life threatening, the less the patient wants to be involved in treatment decisions [24].

To date, few studies investigated psychological characteristics of patients, with some exceptions, which have mostly focused on patients’ personality [27, 28] rather than on situation-specific underlying mechanisms. Furthermore, even fewer studies have examined the psychological reactions to specific contextual variables, such as the individual’s perception of control in the decision-making process.

Active involvement in treatment decision-making requires having sufficient information about one’s illness and available treatment options [20, 29, 30]. However, other studies show that an increasing desire for information not always translates into higher willingness to actively participate in decision-making [18, 23, 24, 26, 31, 32]. This discrepancy between information-seeking and preference to participate has also been reported in qualitative studies with older patients [5]. This contentious pattern demonstrates that the two aspects of patient participation need to be studied as distinct ways of involvement, namely empowerment and health literacy.

### Theoretical framework

The concept of empowerment can refer to the psychological feelings of power, control, and self-esteem. That leads the patient to value autonomy and thus, interest in and desire to participate in healthcare decisions. In this vein, patient empowerment is volitional and doesn’t involve the patient as a passive recipient of information, whose task is the comprehension and acceptance of information. It rather acknowledges the patient as an active processor of information.

An empowered patient extracts self-relevant meaning from proffered information and advice, which he or she subsequently enacts, if believed to be appropriate, in the health situation [33–35]. This subjective perception of control may account for individual differences in the willingness to participate in medical decision-making.

Since previous research suggests that with increasing age, the sense of control and related constructs decline [36, 37], low empowerment may contribute to older patient’s lower preference for participation in medical decision-making.

The patient’s ability to effectively take part in medical decision-making has been the focus of research on health literacy. Health literacy is defined as an individual’s ability “to obtain, process, and understand basic health information and services needed to make appropriate health decisions” [38]. The majority of studies investigating health literacy focused on basic skills, including reading comprehension and numeracy. This is usually referred to as functional literacy [39].

However, Schulz and Nakamoto emphasize that health literacy includes additional elements that go beyond the functional aspects of health literacy [39]. Particularly competencies that foster the derivation of meaning from available information and the use of that information to exercise greater control of and responsibility for one’s health are stressed [39]. These skills might include declarative knowledge (i.e., information about health and medicine) and procedural knowledge (i.e., rules guiding reasoned choice about the proper course of action) [40].

In this sense, if the primary goals of empowerment are efficiency and improved outcomes, then literacy (in all its forms) is an essential foundation. According to Wilson, a patient becomes an expert and an equal actor in the healthcare arena when he or she is well-informed or has access to crucial information regarding his or her own health condition [41]. In this sense, empowerment entails individuals’ ability and motivation to make health-enhancing decisions, which are derived from his or her own knowledge and expertise.

It has to be noted that, while empowerment and health literacy are certainly related, they remain distinct concepts, and it is important to not conflate the two. Studies of health literacy often assume that an informed or expert patient will be empowered in the psychological sense [41]. However, as advanced by the Health Empowerment Model developed by Schulz and Nakamoto, specific attention to literacy and empowerment as independent constructs is critical for improved health outcomes [39].

Since the majority of previous research has focused on describing associations between socio-demographic variables and involvement preferences, the current study aims at better understanding the combined effect of psychological empowerment and health literacy on patients’ participation preferences (ideal involvement) and experiences (actual involvement).

## Methods

### Study population and procedure

The questionnaire was developed in English and back-translated into Italian and German. Pre-tests over three rounds with 5–10 participants in both languages each yielded changes, especially in terms of format, to make the completion of the questionnaire more intuitive. Upon finalization of the questionnaire, ethical approval from the University of Lugano’s Institutional Review Board was obtained. For this cross-sectional study, the sample was recruited in collaboration with the cantonal statistical offices of Lucerne (German-speaking part of Switzerland) and Ticino (Italian-speaking part of Switzerland). Both statistical offices provided a random sample consisting of Swiss residents aged 65 to 80 years old residing in either of the cantons (Ticino *N* = 2500, Lucerne *N* = 1800). Data collection started at the beginning of December 2014 and ended in May 2015. The following documents were sent out via postal mail to each person: a letter stating the purpose of the study with instructions on how to participate, a paper-pencil questionnaire, and a separate form to give informed consent to participate in the study. All information was provided in the language spoken in the region the participant resided. Participants were asked to fill in the self-administered survey, as well as the informed consent sheet, and to mail them back via two separate prepaid envelopes to assure the confidentiality and anonymity of the respondents. Participants who were able to complete and return the survey are considered to have an appropriate level of cognitive ability to take part in the study.

In total, 3633 paper pencil surveys were mailed to the potential participants (i.e., 1833 in Ticino, and 1800 in Lucerne). In order to increase the response rate, prospective respondents were invited to participate in a price draw. The response rate was 23.3% (*N* = 846); 460 respondents came from the Italian-speaking and 386 from the German-speaking region. After a preliminary screening and cleaning of the data, eight surveys were excluded due to limited item response.

The questionnaire included an attentiveness item to evaluate if respondents were devoting sufficient attention to question wording [42]. This item was formulated as following: “The purpose of this question is to assess your attentiveness to question wording. For this question, please mark the response value “1””. The item was placed in the General Self-Efficacy scale, since most of the responses given to this measure were around the extreme value of “4”. This technique allowed identifying non-attentive respondents (23.6% of the full sample). However, due to the high rate of older adults who either did not answer correctly or did not understand the attentiveness question, it was decided to create a less rigorous test of exclusion. It consisted of the following two discarding conditions, of which both had to be fulfilled in order for a participant to be excluded: automatic response pattern (i.e. that a participant selected the same answer for all items of the scale) for (1) the Abbreviated Wake Forest Physician Trust Scale (A-WFPTS) and (2) the “confidence in retrieving specific health information contents scale”. Through this procedure, twelve surveys had to be discarded, yielding a final sample of 826 participants (i.e., 444 completed the Italian survey and 382 the German one).

### Measurements

The questionnaire consisted of validated scales to investigate causes of desired and actual involvement in the decision-making with physician.

Self-reported health status was measured by two single-item sub-scales assessing absolute subjective health on a scale ranging from 1 (*Very bad*) to 5 (*Very good*), as well as comparative subjective health [43]. The latter consisted of the item “Compared to other people of my age and gender, my health status is …” (1 = *significantly worse*, 7 = *significantly better*).

Psychological empowerment was assessed according to Spreitzer’s scale but adapted to the treatment decision-making context [44]. The original scale consists of four sub-dimensions (meaning, competence, self-determination, and impact) with three corresponding items each. The items’ Likert-scales range from 1 (*strongly disagree*) to 7 (*strongly agree*), with higher values suggesting higher levels of empowerment. For brevity, only the self-determination (α = .82) and competence (α = .81) sub-dimensions were included in the survey. As acknowledged by Spreitzer [43], the competence sub-dimension is defined as an individual’s perception of his or her ability to perform tasks with mastery [45, 46]. This component of psychological empowerment closely relates to subjective control (i.e., self-efficacy). However, self-efficacy and psychological empowerment might produce differing and, in some cases, contrasting predictive effects. In order to account for this potential divergence between the two variables, we also measured self-efficacy through the General Self-Efficacy scale developed by Schwarzer and Jerusalem [47]. The scale has been shown to be reliable and one-dimensional across cultures (α = .91) [48].

Health literacy was assessed using three screening questions that were developed and validated by Chew and colleagues [49]. The three items inquired on: (1) the perceived confidence in filling in medical forms; (2) the frequency older adults require help in reading hospital materials; and (3) how often they encounter problems in understanding their medical conditions due to difficulty in reading hospital materials. All items are measured on five-point scales. The first item has response values ranging from 1 (*extremely*) to 5 (*not at all*), whereas items two and three have answer values ranging from 1 (*always*) to 5 (*never*) (α = .72).

Trust in physician, another assessed concept, was elicited according to the Abbreviated Wake Forest Physician Trust Scale (A-WFPTS) that was validated by Dugan and colleagues [50]. The scale is composed of five items scored on a five-point Likert scales ranging from 1 (*strongly disagree*) to 5 (*strongly agree*) (α = .78).

Confidence in retrieving specific health information contents was measured through an adapted version of the National Cancer Institute HINTS’ HC-15A item (α = .88) [51].

Control Preference Scale (CPS-I) by Degner and Sloan was the core dependent variable to elicit respondents’ preferred involvement in treatment decision-making [24]. The CPS consists of a single question with five response statements indicating preference for an active, shared active, collaborative, shared passive, and passive role in decision-making. These were collapsed into three categories for the purposes of this study (active, collaborative, passive). A slightly modified version of the CPS (i.e., different item instructions) was used to establish the perception of the actual involvement participants assumed in the last treatment decision they had to make (CPS-A).

Socio-demographic characteristics included gender, age, marital status, number of children, living situation, cultural background, city of residence, mother tongue, level of education, current and previous occupation, monthly net salary, physician visits per year, presence of chronic condition, and skills in handling computers.

### Statistical analysis

The data was analyzed quantitatively using statistical software SPSS (version 21.0). Prior to main analyses, data was evaluated for uni- and multivariate outliers, non-normality, and missing data. A missing rate of 5% has been applied as cut-off. Since none of the main variables presented more than 5% missing values, missing data management techniques were not applied. Confirmatory factor analysis was performed to evaluate the validity and reliability of the measures. Descriptive statistics were computed in form of absolute values and percentages for all the variables. A correlation matrix was constructed to explore bivariate associations among all the measures. Independent-samples t-tests were conducted to explore associations between socio-demographic (e.g., gender, survey language), independent, and outcome variables. Multinomial logistic regression models were performed to assess the effects of older adults’ psychological empowerment, health literacy, and trust in physician (independent variables) on individuals’ preferred involvement in treatment decision-making and actual involvement experienced (dependent variables). Age, education, and survey language (i.e., applied as a proxy for micro-cultural differences between Swiss-German and Swiss-Italian seniors) were controlled for. Moderation and mediation models were calculated to assess any indirect effect between psychological empowerment and health literacy on the two outcome variables. The Add-on PROCESS macro for SPSS developed by Andrew F. Hayes [52] was used to account for these indirect effects. Moreover, to further corroborate this mediation analysis, we implemented the methodology advanced by Jacobucci [53]. The advantage of that method is that “mediation analyses can now be conducted no matter whether *X*, *M*, and/or *Y* are continuous or categorical” ([53], p. 593). Briefly, the author provides guidance on which method has to be applied (i.e., logistic or linear regression) to compute each single relationship of the model investigated [53]. Five sequential equations have to be fitted in order to establish if mediation has occurred [53]. Our model included one continuous causal variable (*X* = psychological empowerment), one continuous mediator (*M* = health literacy), and one multi-categorical outcome (*Y* = involvement in decision-making). The proposed methods are the following:

1. To estimate the effect of *X* on *M* (path *a*) = > linear regression;
2. To estimate the effects of *X* and *M* on *Y* (path *b*) = > logistic regression.

## Results

Table 1 shows participant socio-demographic characteristics. Most of the older adults declared to be married (72.8%), to live independently (88.5%), to have children (86.4%), to be retired (84.7%), and to visit their physician on average 4.5 times per year.

**Table 1:** Respondent socio-demographics and characteristics

	*N* = 826
Age	
Mean age (SD)	71.8 (4.49)
Range	65-81
Gender	
Male	54%
Marital Status	
Married	72.9%
Living common law	1.7%
Single (never married)	4.2%
Divorced/separated	8.9%
Widowed	12.3%
Living Situation	
Independent	88.5%
Independent with some help by family member	10.2%
Independent with some help by Spitex^a^	0.6%
Assisted living situation	0.5%
Retirement home	0.1%
Children	
Yes	86.4%
Origin/Background	
Swiss German	50.5%
Swiss Italian	34.2%
Swiss French	1.8%
Other	13.4%
Income (monthly net salary)	
Between 0 and 2000 CHF	13.8%
Between 2000 and 4000 CHF	25.8%
Between 4000 and 6000 CHF	18%
Between 6000 and 8000 CHF	12.7%
Above 8000 CHF	10.2%
“I prefer not to answer this question”	19.5%
Education (highest degree completed)	
None	1.6%
Elementary school	3.2%
Middle school	8.8%
High school	12.4%
Professional degree (i.e., apprenticeship)	36.9%
University or professional university degree	32.6%
Other	4.4%
Employment Status	
Retired	84.7%
Physician Visits per Year	
Mean physician visits per year (SD)	4.5 (8.9)
Chronic Disease or any Other Health Condition	
Yes	45.8%

*Note*: ^a^ = Spitex is a subsidized Swiss public homecare service

As reported in Table 2, about 36% of all older adults preferred an active involvement in treatment decisions. Half of the sample expressed preference for a collaborative style in making healthcare decisions while 13.5% wished their doctor to make the final decision for them. One-fifth of the elderly responding to the survey declared to be passive actors in their last treatment decision-making process, followed by almost half (48.1%) of older individuals assuming a shared or collaborative perspective. Nearly one-third (31.1%) of the respondents considered themselves as active treatment decision makers. When comparing older adults’ preferred and actual involvement in treatment decision-making, these were highly corresponding. Individuals reporting a passive involvement in the decision-making with their physician represented the biggest mismatch (i.e., preferred: 13.5% vs. actual: 20.8%).

**Table 2:** Preferred and actual involvement in treatment decisions

Preferred Involvement %	Actual Involvement %			
	Passive	Collaborative	Active	Total
Passive	9.9	2.8	0.7	13.5
Collaborative	8.7	37.3	4.9	50.9
Active	2.2	7.9	25.5	35.6
Total	20.8	48.1	31.1	100

As shown in Table 3, more than two-thirds of the sample can be classified as satisfied with their current involvement in medical decision-making (72.7%). However, the other third of the older adults reported a mismatch between their preferred and actual (or perceived) involvement in treatment decision-making (27.3%). Of those 221 individuals, the majority wished to attain a more active involvement than currently experienced (68.8%), while a minority (31.2%) expressed a preference to take a more passive position than the one actually assumed.

**Table 3:** Difference between preferred and actual involvement in treatment decisions (*N* = 809)

	*N*	%
Wishing a more passive involvement than the actual assumed	69	8.5
Satisfied with current involvement assumed	588	72.7
Wishing a more active involvement than the actual assumed	152	18.8
Total	809	100

In general the sample appeared to be highly empowered (*M* = 5.45, *SD* = 1.23), health literate (*M* = 3.80, *SD* = 0.75), and to assign high levels of trust to their physician (*M* = 4.26, *SD* = 0.72).

Independent-samples t-tests were performed to identify relationships between survey language, gender, chronic disease presence, and the two outcome measures (ideal vs. actual involvement). The results showed significant differences in the involvement levels actually assumed between Swiss-German (*M* = 1.26, *SD* = .67) and Swiss-Italian seniors (*M* = .96, *SD* = .72). Swiss-German respondents assumed higher participation in medical decision-making with their physician, t (810) = −6.14, *p* < .001. The same pattern emerged for the preferred involvement expressed by Swiss-Germans and Swiss-Italians. Indeed, Swiss Germans (*M* = 1.37, *SD* = .60) wished to reach a higher degree of involvement in treatment-related decisions than their Swiss-Italian counterparts (*M* = 1.09, *SD* = .69), t (820) = −6.24, *p* < .001. With regard to gender, there were no significant differences in the assumed and preferred role between male and female participants (*p* = .28, and *p* = .13, respectively). Also the presence of a chronic condition did not discriminate survey respondents in terms of perceived and preferred involvement (*p* = .71, and *p* = .27, respectively). Bivariate correlations to detect any relationship between age, education, physician’s visits per year, and the two outcome variables were conducted. Age proved to be negatively correlated with both dependent measures (CPS-A: *r* = −.17, *p* < .001; and CPS-I: *r* = −.13, *p* < .001), education was positively correlated (CPS-A: *r* = .13, *p* < .001; and CPS-I: *r* = .15, *p* < .001), whereas physician visits per year was not correlated with the two control preference measures.

In terms of micro-cultural differences, independent-samples t-tests were performed to identify significant differences between the two sub-samples with regard to empowerment, health literacy, and trust in physician. It emerged that Swiss-German older adults are more empowered (t (823) = −12.08, *p* < .001) and health literate (t (822) = −4.98, *p* < .001) than Swiss-Italians. Trust in physician appeared to be high in general (*M* = 4.26, *SD* = 0.72), and roughly equal between Swiss-German and Swiss-Italian seniors (*p* = .18).

The predictor variables consisted of psychological empowerment (*M* = 5.45, *SD* = 1.23), health literacy (*M* = 3.80, *SD* = 0.75), and trust in physician (*M* = 4.26, *SD* = 0.72). The two outcome variables were control preference (CPS-A) – actual involvement, and CPS-I – ideal involvement.

The first step of the correlation analysis concerned the bivariate relationships between psychological empowerment, health literacy, trust in physician, and the two dependent variables (CPS-A and CPS-I). Apart from the bivariate correlation between trust in physician and empowerment (*r* = 0.06, *p* = 0.09), all of the five included variables (two dependent and three independent), were mutually correlated (Table 4).

**Table 4:** Pearson correlation coefficients between main variables (*N* = 812)

Variable	1	2	3	4	5
1 Psychological empowerment	--	.47**	.06	.32**	.33**
2 Health literacy		--	.13**	.23**	.19**
3 Trust in physician			--	-.22**	-.17**
4 CPS – actual involvement				--	.64**
5 CPS – ideal involvement					--

*Note*: **p < .001

In a second step, a multinomial logistic regression for predicting health care decision-making involvement (actual and ideal) was run with psychological empowerment, health literacy, and trust in physician selected as the three independent variables. Main co-variates (or adjusting variables) controlled were age, education, and survey language (Italian vs. German).

The final regression model proved to be statistically significant for the CPS – actual involvement (*X* ^2^ (12) = 208.60, *p* < 0.001), and all three predictors significantly improved the regression equation. Pseudo *R* ^2^ statistics showed that the model’s predictors define 23.1% of the variance in the dependent variable (CPS-A) according to Cox and Snell *R* ^2^ statistic, 26.4% according to Nagelkerke *R* ^2^ value, and 12.6% according to McFadden pseudo *R* ^2^ value. The strongest predictive impact was generated by trust in physician (*X* ^2^ (2) = 61.54, *p* < 0.001), followed by psychological empowerment (*X* ^2^ (2) = 52.56, *p* < 0.001), and by health literacy (*X* ^2^ (2) = 6.86, *p* < 0.05). All of the three included co-variates significantly adjusted the main effects of the three predictors. Age detained the strongest predictive force (*X* ^2^ (2) = 24.66, *p* < 0.05), followed by survey language (*X* ^2^ (2) = 9.10, *p* < 0.05), and education (*X* ^2^ (2) = 8.39, *p* < 0.05). Table 5 presents the beta coefficients with their standard errors, ORs and 95% confidence intervals (CIs).

**Table 5:** Beta coefficients (SE), ORs, and 95% confidence interval (CIs) - CPS - actual involvement

	95% CI for Odds Ratio			
	B (SE)	Lower	Odds Ratio	Upper
Collaborative vs. Passive Involvement				
Intercept	6.72 (1.77)***			
Psychological empowerment	.19 (0.09)*	1.01	1.20	1.43
Health literacy	.22 (0.15)	0.93	1.25	1.67
Trust in physician	-.36 (0.17)*	0.50	0.70	0.97
Age	-.08 (0.02)***	0.88	0.92	0.96
Education	.16 (0.09)	0.98	1.17	1.40
Survey language (Italian)	-.64 (0.22)**	0.35	0.53	0.81
Survey language (German)	0	--	--	--
Active vs. Passive Involvement				
Intercept	7.51 (2.12)***			
Psychological empowerment	.80 (0.12)***	1.75	2.22	2.83
Health literacy	.47 (0.18)**	1.12	1.60	2.26
Trust in physician	−1.18 (0.19)***	0.21	0.31	0.44
Age	-.12 (0.03)***	0.84	0.89	0.93
Education	.30 (0.10)**	1.10	1.35	1.65
Survey language (Italian)	-.61 (0.25)*	0.33	0.54	0.88
Survey language (German)	0	--	--	--

*Note: R* ^2^ = .23 (Cox & Snell), .26 (Nagelkerke). Model *X* ^2^(12) = 208.60, *p* < .001. * *p* < .05, ** *p* < .01, *** *p* < .001

The odds ratio showed that as older adults’ empowerment increases by one unit, the change in the odds of being an active player in medical decision-making (rather than a passive player) is 2.22 (95% CI 1.75-2.83). Interestingly, the odds ratio showed that as older adults’ trust in physician decreases by one unit, the change in the odds of being an active player in medical decision-making (rather than a passive player) is 0.31 (95% CI 0.21-0.44). Moreover, the odds ratio showed that as older adults’ health literacy increases by one unit, the change in the odds of assuming an active involvement in healthcare decisions (rather than a passive involvement) is 1.59 (CI 1.12-2.26). In other words, our results showed that an increase in psychological empowerment and health literacy, and a decrease in trust in physician contributed to significantly differentiate the active from the passive group of respondents.

On the other hand, a collaborative involvement (rather than passive) was predicted by psychological empowerment (OR = 1.20, 95% CI 1.01- 1.43), trust in physician (OR = 0.70, 95% CI 0.50- 0.97), age (OR = 0.92, 95% CI 0.88- 0.96), and survey language (OR = 0.53, 95% CI 0.35- 0.81). Health literacy and education did not play a statistically significant role in differentiating the collaborative group of older participants from the passive (reference) group (*p* = .14, and *p* = .08, respectively).

For the other outcome variable (CPS-I), a relatively similar pattern emerged. The final regression model was statistically significant (*X* ^2^ (12) = 171.09, *p* < 0.001), with psychological empowerment, trust in physician, education, age, and survey language as significant predictors of individuals’ ideal involvement. Health literacy did not play a statistically significant role in predicting older participants’ ideal involvement (*p* = .78).

Pseudo *R* ^2^ statistics showed that the model’s predictors explain 19.2% of the variance in the dependent variable (CPS-I) according to Cox and Snell *R* ^2^ statistic, 22.4% according to Nagelkerke *R* ^2^ value, and 10.9% according to McFadden pseudo *R* ^2^ value. In this model the strongest predictor proved to be psychological empowerment (*X* ^2^ (2) = 54.32, *p* < 0.001). Table 6 shows the beta coefficients with their standard errors, ORs and 95% confidence intervals (CIs).

**Table 6:** Beta coefficients (SE), ORs and 95% confidence intervals (CIs) - CPS - ideal involvement

	95% CI for Odds Ratio			
	B (SE)	Lower	Odds Ratio	Upper
Collaborative vs. Passive Involvement				
Intercept	1.85 (2.04)			
Psychological empowerment	.31 (0.10)**	1.12	1.36	1.66
Health literacy	.05 (0.17)	0.75	1.05	1.46
Trust in physician	-.24 (0.19)	0.55	0.79	1.15
Age	-.02 (0.02)	0.94	0.98	1.03
Education	.30 (0.11)**	1.09	1.35	1.66
Survey language (Italian)	-.80 (0.27)**	0.26	0.45	0.77
Survey language (German)	0	--	--	--
Active vs. Passive Involvement				
Intercept	4.44 (2.29)			
Psychological empowerment	0.83 (0.12)***	1.80	2.29	2.92
Health literacy	0.12 (0.19)	0.78	1.13	1.64
Trust in physician	-.89 (0.20)***	0.28	0.41	0.61
Age	-.07 (0.03)**	0.88	0.93	0.98
Education	.45 (0.12)***	1.25	1.57	1.98
Survey language (Italian)	-.84 (0.29)**	0.24	0.43	0.76
Survey language (German)	0	--	--	--

*Note*: *R* ^2^ = .19 (Cox & Snell), .22 (Nagelkerke). Model *X* ^2^(12) = 171.09, *p* < .001. * *p* < .05, ** *p* < .01, *** *p* < .001

In the model focusing on participants’ ideal involvement, the odds ratio showed that as older adults’ empowerment increases by one unit, the change in the odds of being an active player in medical decision-making (rather than a passive player) is 2.29 (95% CI 1.80-2.92). With regard to trust, the odds ratio showed that as older adults’ trust in physician decreases by one unit, the change in the odds of being an active player in medical decision-making (rather than a passive player) is 0.41 (95% CI 0.28-0.61). All three co-variates played a significant role in differentiating the ideal involvement between active and passive decision-makers (see: Table 6). On the other hand, a collaborative involvement was only predicted by psychological empowerment (OR = 1.36 (95% CI 1.12-1.66), education (OR = 1.35 (95% CI 1.09-1.66), and survey language (OR = 0.45 (95% CI 0.26-0.77). Trust in physician and age did not play a statistically significant role in differentiating the collaborative group of older participants from the passive (reference) group (*p* = .22, and *p* = .43, respectively).

To sum up, these analyses confirmed the predictive power of psychological empowerment in significantly differentiating the active and collaborative group of respondents from the passive one (reference category).

A customized/stepwise regression model was conducted in order to identify possible interaction effects between psychological empowerment and health literacy, which is also the hypothesized path according to the Health Empowerment Model [39]. However, these analyses did not show any statistically significant interaction effects.

**Fig. 1:**
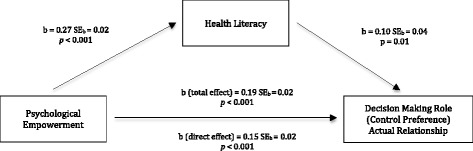
Associations among psychological empowerment, health literacy, and involvement in healthcare decision-making. Note: b: unstandardized effect size coefficients; SE: standard error. Outcomes of mediation analysis model displaying associations between psychological empowerment, health literacy (mediator variable), and involvement in healthcare decisionmaking. Unstandardized effect size coefficients, standard errors, and significance values are indicated. The total effect is the effect of psychological empowerment on the involvement in healthcare decision-making. The direct effect is the effect of psychological empowerment on the involvement in healthcare decision-making when accounting for health literacy

The mediation model, as showed in Fig. 1, introduced health literacy as an intermediary path between psychological empowerment and actual decision-making levels (i.e., CPS-A), while controlling for the influence of trust in physician, age, education, and survey language. The total effect of psychological empowerment on the involvement in decision-making was 0.19 (*p* < 0.001); demonstrating differences in the involvement in decision-making based on psychological empowerment scores. The direct effect of psychological empowerment on involvement in decision-making was 0.15 (*p* < 0.001), showing differences in the involvement in decision-making that resulted from psychological empowerment’s influence on health literacy. The model had an *R* ^2^ of 0.21 (*p* < 0.001) highlighting that psychological empowerment and health literacy explained 21% of the variance in the actual involvement in decision-making, while controlling for the effects of trust in physician, age, education, and survey language. Psychological empowerment significantly differentiated actively from passively involved individuals and this effect was indirectly mediated by health literacy (b = .02, 95% CI 0.01-0.05). In other words, participants with higher health literacy levels assumed a more participatory involvement in their past healthcare decisions with their physician.

To further corroborate this mediation analysis, we followed the procedure introduced by Jacobucci [53]. As shown in Table 7 (second step), the effect of *X* on *M* (path *a*) was: *B* = 0.285; SE = 0.019; *p* < .001.

**Table 7:** Psychological empowerment effect on health literacy (linear regression)

Model		Unstandardized Coefficients		Standardized Coefficients	t	Sig.
		*B*	Std. Error	Beta		
1)	(Constant)	2.251	.105		21.450	.000
	Psychological Empowerment	.285	.019	.467	15.162	.000

*Note*: Dependent variable: health literacy

As specified in Table 5, the effect of health literacy and its standard error on the decision-making role while controlling for psychological empowerment was (path *b*): *B* = 0.47; SE = 0.18; *p* < .01. By computing the *Z* (*B*/SE) value of path *a,* we obtain: *Za* = 15; whereas the *Z* value of path *b* equals: *Zb =* 2.611. Their product (*Z* = *a* * *b*) yielded a value of 39.166, and their collected standard error ($\sqrt{Z_a^2+{Z}_b^2+1}$) was 15.258. The final equation, which creates the *Z*_*Mediation*_ value, yielded a coefficient of 2.566. As stated by Jacobucci [53], the coefficient *Z*_*Mediation*_ “is significant at the α = 0.05 level if it exceeds 1.96” (p. 593). In light of this additional analysis, we can conclude that the significant indirect effect obtained by Hayes’ PROCESS macro [52] is confirmed by Jacobucci’s method [53].

## Discussion

### Summary of main findings

The current research, to our knowledge, is the first attempt that investigates the combined role of psychological empowerment and health literacy on older adults’ participation preferences in treatment decision-making with their doctor. In particular, we examined and compared the ideal (or preferred) and actual (or perceived) involvement in treatment decision-making of 826 older adults residing in two different language regions of Switzerland. Most of the Swiss older adults surveyed (72.7%) were satisfied with the current decision involvement level assumed with their physician. Interestingly, the majority of senior respondents preferred at least some participation when facing medical decisions (active decision-making: 35.6% + collaborative decision-making: 50.9% = 86.5%). With regard to the actual (or perceived) involvement in medical decision-making; psychological empowerment, health literacy, low levels of trust in physician, education, younger age, and completing the survey in German significantly contributed to differentiate actively involved seniors from passive respondents. Intriguingly, health literacy proved also to be a significant mediating force of the relationship between empowerment and the outcome variable (CPS-A), even after controlling for the effects of trust in physician, education, age, and survey language (Italian vs. German).

With regard to the ideal (or preferred) involvement in medical decision-making (CPS-I), the analyses showed that psychological empowerment, low levels of trust in physician, education, younger age, and survey compilation in German significantly contributed to differentiate active seniors from passive respondents. In this specific case, health literacy did not significantly contribute to the conceptualized model (neither as pure predictor, nor as mediating variable).

### Older Adults’ decision making role

Our data showed that a substantial part of Swiss older adults were satisfied with the degree of involvement that they assumed in treatment decisions taken with (or by) their physicians (72.7%). This data is consistent with the study conducted by Voegel, Helmes and Hasenburg [54].

Overall 86.5% of our respondents preferred some participation in treatment decision-making (active decision-making: 35.6%, collaborative decision-making: 50.9%). This finding on one hand contrasts with past research investigating older patients’ preferences (e.g., [5, 11, 13, 55]) in comparison with their actual involvement in treatment decisions, (e.g., [56–58]). On the other hand, the research conducted in Switzerland by Wang and Schmid (sample age: 15+ years) reported concordant estimates of the high rate of Swiss citizens preferring to assume an active (or collaborative) involvement in medical decisions, even when comparing different regional areas (German- vs. Italian-speaking) [59].

### Health literacy, psychological empowerment, and role in decision-making

Consistent with our expectations, psychological empowerment played a significant role in differentiating older adults preferring and assuming an active or collaborative involvement in health care decision-making with their physician as compared to the reference group that favored passive involvement. However, health literacy only predicted the actual involvement in the past treatment decision, significantly differentiating the active from the passive involvement group.

Previous studies assessing associations between health literacy and treatment decision-making mainly focused on the effects of health literacy and the information exchange in the decision making process [60]. In almost all the studies reviewed by Malloy-Weir and colleagues, findings concerning the relationships between health literacy and measures of decision-making (for all three decision-making stages (as conceptualized by Charles, Gafni, & Whelan [3]) were contradictory and “not always positively associated” [60].

Interestingly, our results showed that health literacy mediates the relationship between psychological empowerment and the actual involvement individuals assumed with their physician in the past treatment decision. As argued by Schulz and Nakamoto “health literacy and patient empowerment are distinct concepts but closely interwoven and must be considered in conjunction to understand individual health behavior […]” [39]. Our current findings are best interpreted according to their conceptual model, which identifies four different patient behavior styles based on health literacy and psychological empowerment. The model labels individuals who score high on health literacy and empowerment as “effective self-managers”. In our sample, this group most likely assumed an active decision-making style in their last treatment decision.

In light of this, we can conclude that empowerment is a sufficient prerequisite for the intention to attain an active or collaborative role in future healthcare decisions. However, for this preference to be translated into actual behavior, an adequate level of health literacy is also required to enable “the consumer to make informed and reasoned choices” [39]. Hence, our results find a potential justification of the different effects (direct and indirect) generated by our two independent on the two outcome variables (i.e., CPS-A, and CPS-I).

The significant correlation between health literacy and patient empowerment, coupled with what we have found in the regression analyses (i.e., health literacy mediated the relationship between empowerment and actual involvement), supports the circle of researchers who claim that health literacy is the empowering force of patients [61–64]. Whether these findings are compatible with other older adult populations (especially patients with a specific disease) has to be investigated in further research.

### Trust in physician and role in decision-making

Most of our respondents attributed high levels of trust to their physician. Moreover, the findings of the present study showed that as patients’ trust in their physician increases, the preference and the actual experience of involvement decreases. The research conducted by Kraetschmer and colleagues [65], showed concurrent results matching the present study’s findings. Nonetheless, past evidence on the relationship between trust in physician and decision-making role has been controversial [66–69]; also cited in [65]. Since trust may be a fundamental component of involvement in treatment decisions [65], future studies are encouraged to foster further evidence on the exact influence of trust in physician on the willingness to participate in medical decisions (i.e., barrier or facilitator).

### Health literacy levels in the Swiss context

This study also presents new findings on health literacy levels in Switzerland and among two of its language groups. The results of this study concur with studies conducted in Switzerland and in the US [49, 70]. Indeed, the results of this research are in line with the findings from a study that was earlier conducted in Switzerland [70], where varying health literacy levels were found between Swiss German and Swiss Italian sub-samples. Although Swiss-Germans in the present study were slightly less literate than in Connor and colleagues’ study [70], both studies report that Swiss-Germans are slightly more health literate than Swiss-Italians. These differences in health literacy levels between the two Swiss-German sub-samples might be explained by two reasons: (1) different measures of health literacy and (2) different mean ages of the samples.

### Socio-demographic characteristics and decision making role

The findings on several of the assessed socio-demographic characteristics (i.e., age, education, and survey language) were in line with former research (e.g., [5, 14, 15, 18, 19]), explaining the variation in the perceived and desired role older adults assume in treatment decision-making. However, with regard to age-related differences, as acknowledged by several researchers [71, 72], cross-sectional designs may overestimate age-related differences “due to cohort effects” ([71], p. 17). As a matter of fact, such research designs are unable to establish if the effects occurred “due to developmental age processes or to shared experiences characterizing cohort effects” ([71], p. 17). Additionally, past research found that the severity of one’s illness [23, 24, 26], the type of decision, as well as the knowledge level required to make that choice [73] were all crucial factors determining an individual’s willingness to engage in shared decision-making. Patients whose condition had recently worsened were more likely to disengage from decision-making [74]. It is therefore imperative to specify that the majority of the respondents in this study were not experiencing any life-threatening illness (e.g., cancer), but some suffered from chronic morbidities (45.8% of the sample). This provides some explanation for the tensions between the present and past findings about individuals’ willingness to assume an active role in treatment decision-making.

For example, in a systematic review conducted by Tariman and colleagues, twenty out of the twenty-two evaluated studies applied the CPS scale to assess cancer patients’ desired and/or actual involvement in decision-making [75]. Even if in only five of these twenty-two studies the patients’ preferred involvement was concordant with our findings [76–80], the overall trend showed that cancer patients tend to prefer a less collaborative involvement compared to a mixed sample like ours.

Nevertheless, our result might reflect older adults’ willingness to adjust their decision-making style to the socio-political change promoting patient autonomy, or the so-called patient-centered approach [10].

### Actual and preferred decision making role

In a recent qualitative study conducted by Tariman and colleagues, 55% of older newly diagnosed myeloma patients expressed a shared decision-making involvement as the ideal one (collaborative), while 40% wished to assume an active decision-making style [81]. These findings match with ours, which report patients’ preferred involvement in treatment decisions.

Consistent with previous findings, the current study also reported a mismatch between desired and actual (or perceived) involvement in the treatment decision-making process (for a review consult: [75]). The greatest discrepancy between preferred and actual involvement was observed for the group wanting to assume a passive involvement. Indeed, from the passive recipients of care, who represented one fifth of the participants (20.8%), only 13.5% of the final sample really wished to assume a non-participatory involvement in future treatment decisions (i.e., actual vs. desired involvement). The majority of older adults reporting a misalignment between preferred and actual involvement declared a preference to attain a more active involvement than the one originally assumed (*N* = 173). This trend is consistent with the studies reviewed by Tariman and colleagues [75]. A non-negligible 10% of the elderly population wished to play a less collaborative involvement than the one currently assumed.

### The control preference scale (CPS)

This study advances the field by adding knowledge on the psychosocial antecedents of patients’ involvement in healthcare. A study assessing the validity of different outcome measures applied in medical decision-making concluded that the CPS is one of the few scales that satisfied “high appraisal standards of quality” ([82], also cited in [83], p. 5). The CPS scale, a widely used measure among researchers in the area of medical decision-making (e.g., [54, 75, 82]) was used to elicit older adults’ participation preferences and actual experiences in treatment decisions. Its application to the present context proved to be fruitful.

### Limitations

A first limitation of this study concerns the generalizability of the findings to the Swiss elderly population. Our sample, based on a random draw by two cantonal statistical offices, represented only older adults aged 65 to 80 years old who reside in the Italian and the German-speaking regions of Switzerland. We did not collect data from the French-speaking area. In light of this, our findings are more likely to be applicable and genuine to the geographical areas of Switzerland where Italian and German are the languages in use. Past research emphasized that differences in culture might explain the dissimilarities in willingness to engage in medical decisions [54], which our data suggests as well. According to census data [84, 85], our sample seemed to be partially representative of the whole older adults’ population residing in Switzerland. Indeed, the marital status of our sample concurred with the entire Swiss population of older adults [85]. Small differences can be reported regarding gender; our sample consisted of 54% male respondents, whereas the Swiss population aged 65 to 80 years old consists of 53.3% women [84]. According to estimates computed by the Swiss Statistical Office [84], the percentage of individuals who completed primary education and those who attained a university or applied university degree are reverted in our sample compared to the census date, while our sample corresponded for those completing a secondary education degree. In fact, our sample resulted to be more educated than the senior population residing in the country [84]. Because of the self-administration format of our survey, it can be presumed that Swiss residents who had higher educational levels than the general elderly population were more prone to complete the questionnaire. This homogeneous range of educational levels among our respondents might have hindered the generalizability of our results. Nonetheless, it has to be noted that the older adults participating in our survey represented a probabilistic random sample, thereby mitigating possible sampling bias.

A second limitation poses the single-item outcome measure to elicit the actual involvement assumed in the past treatment decision, which was an adaptation of Degner and Sloan’s participation preference item [24]. The main item modification for preferred versus actual involvement in treatment decision-making concerned only the instructions provided to introduce the question. As the original item wording uses the term “prefer” in the present tense for both (CPA-A and CPS-I), some participants may not have properly understood the difference between the two similar items. Further research using the CPS scale to assess both preferred and actual involvement should take care of this issue, either by modifying the tense of the verb from the present to the past, or by avoiding the use of the verb “to prefer” in the actual involvement items: e.g., “*I prefer to leave all decisions regarding treatment to my doctor*” (CPS scale, passive involvement response used in the present survey) to “I leave all decisions regarding my treatment to my doctor” (passive involvement response suggested for future surveys with the CPS-A scale). The wording of the questions in this study may not have allowed a sufficient differentiation between older adults’ actual and preferred role in treatment decision-making due to recall effects (i.e. that the participants remembered the similar question wording from the first question about their actual involvement when answering the second question about their preferred involvement in treatment decision-making). To avoid similar issues in future research, scholars should consider remaining closer to the method of Degner and colleagues [86], where individuals were prompted by a researcher to order vignettes that showed different levels of involvement based on their own preferences. However, in a large-scale quantitative survey (as in this study), that is not feasible.

Third, as our outcome measures relied on single items rather than on multi-item techniques, our patients’ involvement estimates have to be considered with caution. The usage of self-report measures to assess the levels of health literacy, empowerment, trust in physician, and patients’ involvement in treatment decisions is potentially subject to recall and social desirability bias. Future studies assessing the validity of these self-reported variables might triangulate data and include measures that elicit doctors’ perceptions of the patient involvement and physicians’ interpersonal style (paternalistic vs. mutual, see [87]).

While we assumed that respondents who completed the survey have an appropriate level of cognitive ability to understand the survey questions, we have not assessed this construct in statistical or formal terms. Since it was an auto-administered questionnaire to a random sample, we could not verify whether participants completed the questionnaire by themselves and independently. Nonetheless, this limitation may be unavoidable in all self-administered survey designs that omit objective cognitive status measures.

Furthermore, longitudinal designs are called for in future research, as our static assessment of involvement in treatment decision-making did not permit a verification of preference and actual behavior change over time – either gradually or more suddenly as a reaction to a specific event [13].

### Implications for research and practice

The current study provides empirical evidence on the interrelated effects of health literacy and psychological empowerment toward older adults’ participation in healthcare decisions with their physician. Further efforts are needed to validate the findings of this study in other (more specific) health contexts and elderly populations. To deepen the empirical evidence of the results obtained, the application of other measures to assess the concepts included in this study (i.e., health literacy, psychological empowerment, trust in physician, and involvement in decision-making) is warranted.

The benefits on health outcomes for individuals playing a responsible and active involvement in healthcare decisions are un-debated [59, 88–92], and geriatricians are recommended to make their patients aware of that positive relationship. However, it is also important to respect individuals’ preferences of involvement; seniors who wish to completely delegate decisions to their healthcare providers should still be able to do so. Since the majority of Swiss older adults aim to assume an active or collaborative involvement in treatment decisions, medical professionals are encouraged to present easy-to-understand information about the available treatment options and related benefits/risks of those choices (i.e., a fundamental prerequisite for participation).

## Conclusions

The results of this study show that both health literacy and patient empowerment affect elderly persons’ actual involvement in medical decision-making. Due to higher reported levels of psychological empowerment and health literacy, Swiss-German significantly preferred and assumed higher participation in medical decisions than Swiss-Italian seniors. Trust in physician and age appeared to be barriers to involvement, whereas education served as a facilitator.

As older adults’ health literacy plays a mediating role in individuals’ active involvement in health care decision-making, public health efforts should aim at developing programs and appropriate information that facilitate this process especially for seniors with moderate or lower levels of health literacy. The current investigation showed that adequate health literacy levels are essential (but not sufficient) in order to reach higher rates of participation in the healthcare context. This research complements past evidence by adding knowledge on the psychosocial antecedents and their combined effects on patients’ involvement in healthcare.
